# The Antipsychotic Risperidone Alters Dihydroceramide and Ceramide Composition and Plasma Membrane Function in Leukocytes In Vitro and In Vivo

**DOI:** 10.3390/ijms22083919

**Published:** 2021-04-10

**Authors:** Alberto Canfrán-Duque, Óscar Pastor, David García-Seisdedos, Yessenia L. Molina, Bohdan Babiy, Milagros Lerma, Carmen Sánchez-Castellano, Javier Martínez-Botas, Diego Gómez-Coronado, Miguel A. Lasunción, Alfonso J. Cruz-Jentoft, Rebeca Busto

**Affiliations:** 1 Servicio de Bioquímica-Investigación, Hospital Universitario Ramón y Cajal, IRyCIS, 28034 Madrid, Spain; Alberto.Canfran-duque@nyulangone.org (A.C.-D.); dgseis@hotmail.es (D.G.-S.); yesse1497@gmail.com (Y.L.M.); mila93.lerma@gmail.com (M.L.); javier.botas@hrc.es (J.M.-B.); diego.gomez@hrc.es (D.G.-C.); malasuncion@hotmail.com (M.A.L.); 2 Servicio de Bioquímica-Clínica, Hospital Universitario Ramón y Cajal, IRyCIS, 28034 Madrid, Spain; oscar.pastor@salud.madrid.org (Ó.P.); bohdan@hotmail.es (B.B.); 3 CIBER Fisiopatología de la Obesidad y Nutrición (CIBERobn), Instituto de Salud Carlos III (ISCIII), 28029 Madrid, Spain; 4 Servicio de Geriatría, Hospital Universitario Ramón y Cajal, IRyCIS, 28034 Madrid, Spain; carmen.sanchezc@salud.madrid.org

**Keywords:** antipsychotic, risperidone, older person, lipid metabolism, sphingolipid, lysophospholipid

## Abstract

Atypical or second-generation antipsychotics are used in the treatment of psychosis and behavioral problems in older persons with dementia. However, these pharmaceutical drugs are associated with an increased risk of stroke in such patients. In this study, we evaluated the effects of risperidone treatment on phospholipid and sphingolipid composition and lipid raft function in peripheral blood mononuclear cells (PBMCs) of older patients (mean age >88 years). The results showed that the levels of dihydroceramides, very-long-chain ceramides, and lysophosphatidylcholines decreased in PBMCs of the risperidone-treated group compared with untreated controls. These findings were confirmed by in vitro assays using human THP-1 monocytes. The reduction in the levels of very-long-chain ceramides and dihydroceramides could be due to the decrease in the expression of fatty acid elongase 3, as observed in THP-1 monocytes. Moreover, risperidone disrupted lipid raft domains in the plasma membrane of PBMCs. These results indicated that risperidone alters phospholipid and sphingolipid composition and lipid raft domains in PBMCs of older patients, potentially affecting multiple signaling pathways associated with these membrane domains.

## 1. Introduction

Antipsychotic drugs are widely used to manage psychiatric disorders. These drugs are classified into typical or first-generation antipsychotics (FGAs), atypical or second-generation antipsychotics (SGAs), and third-generation antipsychotics. The therapeutic efficacy of the first two classes is strongly associated with the antagonism of dopamine D2/D3 receptors [1]. SGAs have a lower affinity for D2/D3 receptors and a higher affinity for serotonergic, histaminergic, muscarinic, and adrenergic receptors than FGAs [1]. Third-generation antipsychotics act via partial agonism at D2/3 receptor sites [1]. 

FGAs and SGAs cause serious side effects, including acute and chronic extrapyramidal symptoms and movement disorders [1]. SGAs cause fewer neurological side effects but block the receptors of other neurotransmitters, which is thought to be related to other adverse effects [1,2]. SGAs are widely used in the treatment of psychosis and behavioral problems in older patients with dementia because these drugs are safer than FGAs [2]. However, SGAs are potentially associated with an increased risk of stroke and death in such patients [3,4,5]. The mechanisms underlying the link between stroke and SGAs are incompletely understood [6].

Exposure to antipsychotics has been shown to alter lipid homeostasis [7,8]. Both FGAs and SGAs inhibit cholesterol biosynthesis in vitro by decreasing the activity of enzymes involved in this pathway, resulting in the accumulation of different sterol intermediates [7,9,10,11]. This alteration in sterol composition affects lipid raft formation and consequently hormone signaling, as demonstrated for insulin and somatostatin in cells treated with the FGA haloperidol [9,10]. Furthermore, antipsychotics impair intracellular trafficking of cholesterol by interfering with cholesterol egress from endolysosomes, thereby reducing the transport of endocytosed LDL cholesterol to the endoplasmic reticulum [7,10,11,12] and trans-Golgi [13]. As cationic amphiphilic drugs (CAD), antipsychotics alkalinize lysosomes, affecting lysosomal function, as demonstrated for haloperidol [14]. Moreover, antipsychotics increase LDL receptor transcription, thus stimulating LDL endocytosis and worsening the intracellular accumulation of LDL-derived lipids [7,8].

Given the critical roles of lipids in cellular mechanisms, lipid analysis may help understand the efficacy and side effects of antipsychotic treatments. Several studies have assessed the role of phospholipids and sphingolipids in the structure and function of cellular membranes in schizophrenia before and after antipsychotic treatment [15,16,17,18,19]. Ceramides and other sphingolipids are the main components of lipid rafts in plasma membranes, where they act as structural and signaling molecules [20]. However, to the best of our knowledge, the effects of antipsychotic treatment on membrane lipid composition in older persons have not been examined.

This study evaluates the effects of risperidone treatment on phospholipid and sphingolipid composition and lipid raft function in peripheral blood mononuclear cells (PBMCs) of older patients with dementia compared with patients not receiving antipsychotic. The results showed that risperidone decreased the levels of dihydroceramides, very-long-chain ceramides, and lysophosphatidylcholines in treated patients. Moreover, this drug disrupted lipid raft domains in the plasma membranes of PBMCs. These results indicate that risperidone alters the phospholipid and sphingolipid composition and impairs lipid rafts in PBMCs of older persons, and these changes can potentially affect multiple signaling pathways. 

## 2. Results

### 2.1. Patients Characteristics

Twenty patients older than 70 years were included in the analysis. Of these, 10 participants did not receive antipsychotic treatment (Control group), while the other 10 were risperidone-treated patients that had reached a stable dose of this medication at least 3 months before the recruitment (Table 1). The mean daily dose of risperidone in the treated group was 1.05 ± 0.15 mg. Age, sex distribution, and co-medications were similar between the two groups (Table 1).

The patients in both groups were allowed to eat whatever they wanted and were not placed on a restricted diet or enteral nutrition (Table 1). Only one risperidone-treated patient received oral supplements. Most of the treated patients needed self-care eating, while in the control group six of them needed self-care eating (Table 1).

There were no significant intergroup differences in the levels of biochemical markers, except for ALT and AST (Table 2). Although ALT and AST values were within the normal range, the means were significantly higher in treated patients than in the controls.

### 2.2. Phospholipid and Sphingolipid Composition in PBMCs

**Figure 1:**
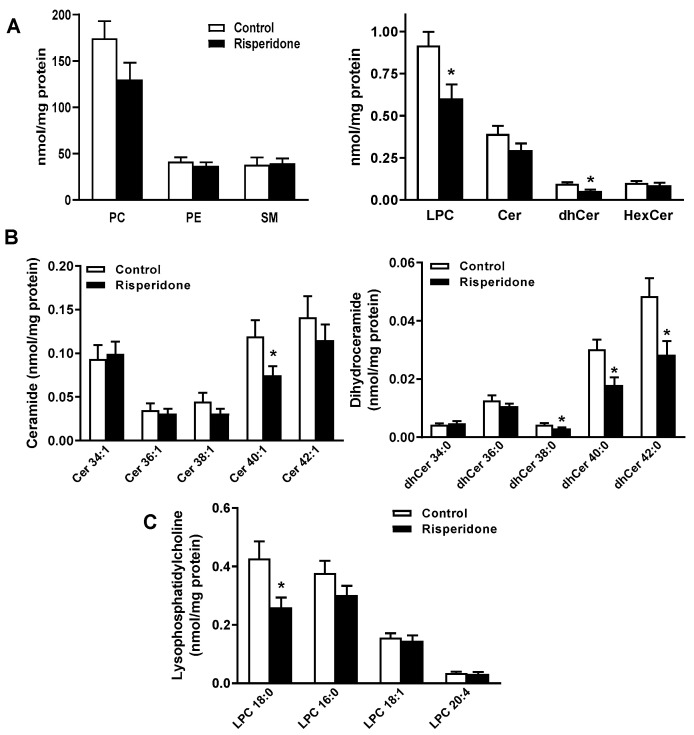
Phospholipid and sphingolipid contents in PBMCs of risperidone-treated older patients and untreated controls. (**A**) Lipid class levels (nmol/mg of protein) of phosphatidylcholine (PC), phosphatidylethanolamine (PE), lysophosphatidylcholine (LPC), sphingomyelin (SM), hexosylceramide (HexCer), ceramide (Cer), and dihydroceramide (dhCer) were measured by mass spectrometry. (**B**) Ceramide and dihydroceramide species. (**C**) Lysophosphatidylcholine species. Results are means ± SEMs of ten samples per group. Statistical comparisons shown are risperidone vs. control (* *p* < 0.05).

The levels of phospholipids (phosphatidylcholine, phosphatidylethanolamine, and lysophosphatidylcholine) and sphingolipids (ceramide, dihydroceramide, hexosylceramide, and sphingomyelin) were measured in PBMCs from the control and risperidone-treated older patients (Figure 1, Figure S1 and Table S2). There were no significant differences in the levels of phosphatidylcholine, phosphatidylethanolamine, ceramide, hexosylceramide, and sphingomyelin between the groups (Figure 1A). However, the concentrations of lysophosphatidylcholine and dihydroceramide were significantly lower in the risperidone-treated group (Figure 1A). Moreover, the ceramide/dihydroceramide concentration ratio was significantly lower in the control patients compared with the risperidone-treated group (control group = 4.092 ± 0.360 (mean ± SEM, *n* = 10) and risperidone-treated group = 5.749 ± 0.448 (*n* = 10) (*p* < 0.01)).

The levels of very-long-chain ceramides (Cer 40:1) and dihydroceramides (dhCer 38:0, dhCer 40:0, or dhCer 42:0) and lysophosphatidylcholine species with 18:0 acyl chains were significantly lower in the risperidone-treated patients (Figure 1B,C). 

Small changes were observed in some species of phosphatidylethanolamine and sphingomyelin in treated patients compared with the control ones (Figure S1 and Table S2), the most outstanding being the increases in PE 34:2, PE 36:3, and SM 36:2. There were no significant differences in the levels of phosphatidylcholine and hexosylceramide species between the groups (Figure S1 and Table S2).

### 2.3. Phospholipid and Sphingolipid Composition in Human Monocyte THP-1 Cells

**Figure 2:**
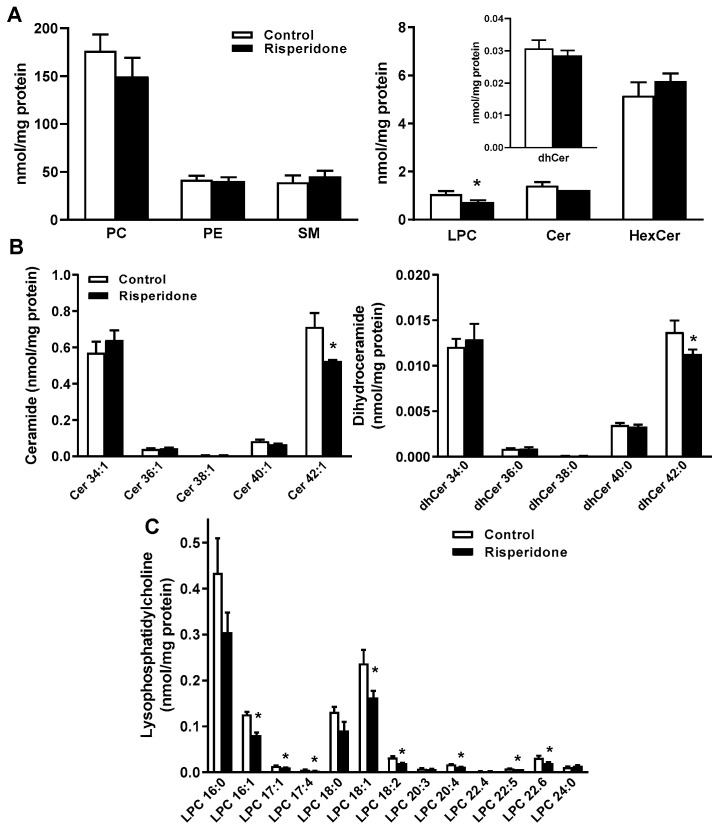
Effects of risperidone treatment on the phospholipid and sphingolipid profiles in monocyte THP-1 cells. Cells were treated or not with 25 µM risperidone for 72 h. (**A**) Lipid class levels (nmol/mg of protein) of phosphatidylcholine (PC), phosphatidylethanolamine (PE), lysophosphatidylcholine (LPC), sphingomyelin (SM), hexosylceramide (HexCer), ceramide (Cer), and dihydroceramide (dhCer) were measured by mass spectrometry in risperidone-treated and control cells. (**B**) Ceramide and dihydroceramide species. (**C**) Lysophosphatidylcholine species. Results are means ± SEMs of three independent experiments. Statistical comparisons shown are risperidone vs. control (* *p* < 0.05).

We sought to investigate whether the decrease in the levels of very-long-chain ceramides and dihydroceramide in PBMCs of risperidone-treated patients also takes place in the human monocyte cell line. THP-1 monocytes were treated or not with 25 µM risperidone for 72 h in LPDS-supplemented medium, and the levels of phospholipids (phosphatidylcholine, phosphatidylethanolamine, and lysophosphatidylcholine) and sphingolipids (ceramide, dihydroceramide, hexosylceramide, and sphingomyelin) were measured (Figure 2, Figure S2 and Table S3). There were no significant differences in the concentrations of phosphatidylcholine, phosphatidylethanolamine, sphingomyelin, ceramide, dihydroceramide, and hexosylceramide between risperidone-treated and control cells (Figure 2A). However, lysophosphatidylcholine levels were significantly lower in risperidone-treated cells (Figure 2A). 

The concentrations of Cer 42:1 and dhCer 42:0 species and some lysophosphatidylcholine species were significantly lower in risperidone-treated cells (Figure 2B,C). 

There were no significant differences in the concentrations of hexosylceramide and sphingomyelin species between treated and control THP-1 cells (Figure S2 and Table S3). Quantitatively small changes were observed in some species of phosphatidylcholine and phosphatidylethanolamine between treated and untreated THP-1 cells (Figure S2 and Table S3). 

Interestingly, the levels of very-long-chain ceramides, dihydroceramides, and lysophosphatidylcholines were significantly lower in risperidone-treated monocyte cells, recapitulating our findings in PBMCs.

### 2.4. Risperidone Disrupts Lipid Rafts (Detergent-Resistant Membranes [DRMs]) in PBMCs and Monocyte THP-1 Cells

We previously reported that haloperidol changed the lipid composition of lipid rafts and the function of these domains in different cell lines, as indicated by the alteration of signal transduction processes [9,10]. 

CD13 localizes to detergent-resistant lipid rafts in monocytes [21]. The flow cytometric assay described by Gombos et al. [22] was used to assess whether risperidone treatment affected the association of CD13 with DRMs in PBMCs. This assay has been validated in a number of cell types, including primary monocytes, T cells, B cells, and various cell lines [21,22,23]. This assay estimates the FCDR index. A value close to 0 is obtained if a surface protein is highly soluble after detergent treatment (i.e., does not localize to DRM) and a value close to 1 represents an insoluble surface protein after detergent treatment (i.e., localizes to DRM). The FCDR index values of CD13 in PBMCs from control and treated patients were 0.805 ± 0.127 (mean ± SEM, *n* = 10) and 0.472 ± 0.045 (*n* = 10) (*p* < 0.05), respectively, indicating the strong association of this surface antigen to lipid rafts in the control patients and a decrease in the association with lipid raft or DRM domains in risperidone-treated patients. This indicates that PBMCs of risperidone-treated patients have altered lipid raft domains.

The FCDR index values of CD13 in risperidone-treated and control THP-1 cells were 0.572 ± 0.104 (*n* = 4) and 0.832 ± 0.063 (*n* = 4) (*p* < 0.05), respectively, which is consistent with the results in PBMCs of older patients.

### 2.5. Effects of Risperidone on the Gene Expression of Fatty Acid Elongases

**Figure 3:**
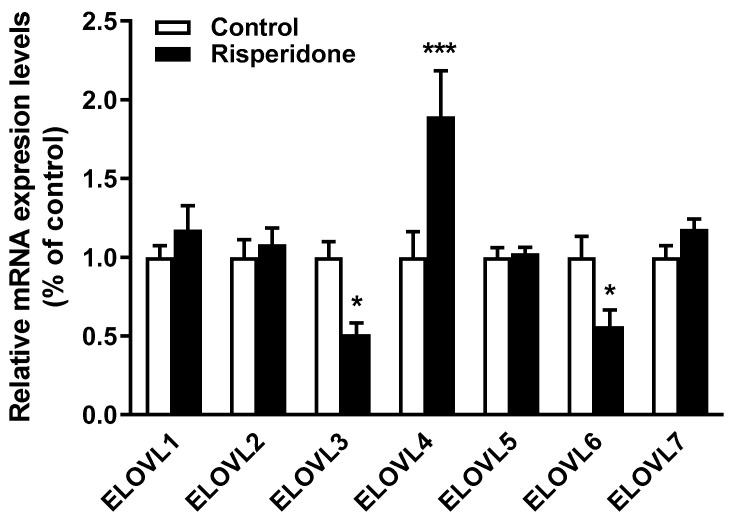
Effects of risperidone on the gene expression of fatty acid elongases (ELOVLs). THP-1 cells were treated or not with risperidone for 72 h. Cells were collected, total mRNA was extracted, and mRNA expression was quantified by qRT-PCR. Expression levels were normalized to RPLP0 and are shown as percentages of control. Data are means ± SEMs of five independent experiments performed in triplicate. Statistical comparisons shown are risperidone vs. control (* *p* < 0.05 and *** *p* < 0.001).

EVOVLs are fatty acid elongases involved in the synthesis of ceramides with long-chain fatty acids [24]. The mRNA levels of different elongase isoforms (ELOVL1-7) were measured in THP-1 cells. ELOVL3 and ELOVL6 expression decreased, whereas ELOVL4 expression increased in risperidone-treated cells (Figure 3). There were no significant differences in the expression levels of ELOVL1, ELOVL2, ELOVL5, and ELOVL7 between treated and control cells (Figure 3).

## 3. Discussion

This study demonstrates that a 3-month risperidone treatment in older patients alters the phospholipid and sphingolipid composition of circulating PBMCs and disrupts detergent-resistant or lipid raft domains in the plasma membrane. These changes can affect signaling pathways associated with these membrane domains and may play a role in the pathophysiology of cardiovascular side effects of this drug.

Persons older than 70 years (mean age of 88.5 years) were included in this study. Risperidone at a daily dose of 1.05 ± 0.15 mg for at least 3 months was well tolerated. Plasma ALT and AST levels increased slightly in treated patients, which may be attributed to drug metabolism in the liver, but remained within the normal range [25].

We studied the levels of phospholipid and sphingolipid in PBMCs isolated from risperidone-treated patients and controls. With regard to sphingolipids, this drug caused no detectable changes in the concentrations of hexosylceramide, sphingomyelin, and ceramide but significantly decreased dihydroceramide levels. Moreover, risperidone significantly decreased the levels of ceramide and dihydroceramide species with very-long-chain fatty acids in PBMCs. The decrease in very-long-fatty acid ceramide and dihydroceramide species was confirmed by in vitro assays using human THP-1 monocytes treated with risperidone. Lipidomic studies on psychotic states and the effects of drug treatment are scarce, and the results are contradictory in some respects. Yan et al. have demonstrated that ceramide and glucosylceramide species in plasma are relevant in schizophrenia and that antipsychotic treatment dysregulates several lipid species [18]. Smesny et al. reported a decrease in total ceramide levels in the skin of patients suffering from first-episode schizophrenia [26]. In contrast, total ceramide levels increased in the skeletal muscle of SGA-treated patients compared with those in the mood stabilizer group [16]. In preclinical studies, mice chronically treated with haloperidol showed a decline in brain sphinganine, a lipid intermediate in ceramide synthesis [27]. In female rats, clozapine treatment significantly decreased ceramide and sphingomyelin levels in the liver, whereas olanzapine increased sphingomyelin levels [17]. However, these SGAs did not alter sphingolipid levels in the skeletal muscle [17]. The present study is the first carried out in patients over 70 years, and the results showed that risperidone decreased the concentration of very-long-chain ceramides and dihydroceramides and in total dihydroceramide in peripheral tissues such as PBMCs. The extent to which these changes in sphingolipid levels during antipsychotic treatment are markers for the therapeutic action or are related to side effects is currently unclear.

Ceramides and other sphingolipids have structural and signaling functions in cell membranes [20]. Studies on long-chain ceramides and very-long-chain (≥C22) ceramides revealed that membranes containing the latter are more tightly packed and interdigitation with other lipids is increased compared with membranes with long-chain (<C22) ceramides [28,29]. In hepatocytes, the depletion of C22:0-C24:0 ceramides increased plasma membrane fluidity [28] and the elevation of dihydrosphingolipid levels increased membrane rigidity [30]. Therefore, the decrease in dihydroceramide and very-long-chain ceramide levels may decrease membrane rigidity in PBMCs of risperidone-treated patients.

Ceramides have been associated with disrupted insulin signaling and insulin resistance [31]. Park et al. demonstrated that a reduction in the levels of very-long-chain ceramides decreased insulin receptor phosphorylation in the liver and inhibited insulin receptor translocation into DRM, leading to insulin resistance [32]. Our results showed that risperidone significantly decreased the FCDR index of CD13 in PBMCs, which agrees with the results found in human monocyte THP-1 cells and indicates that risperidone alters lipid rafts or DRMs in cell membranes. Our previous studies showed that haloperidol treatment disrupted lipid rafts in several cell lines and affected insulin and somatostatin signal transduction [9,10]. These results suggest that risperidone treatment may affect certain signaling pathways associated with lipid rafts, a hypothesis that needs to be elucidated.

Ceramide metabolism is closely connected to fatty acid metabolism. Fatty acids longer than 16 carbons are required for the synthesis of long-chain ceramides and are produced by seven ELOVL isoforms [24]. Trying to explain the decrease of very-long-chain ceramides and dihydroceramides, we explored the mRNA expression of ELOVL enzymes responsible for fatty acid elongation in cultured monocytes. We found that risperidone decreased ELOVL3 and ELOVL6 and increased ELOVL4 mRNA levels. ELOVL3 elongates both saturated and unsaturated C16-C22 fatty acids and ELOVL6 elongates shorter fatty acids (C12-C16) [24]. The decreased expression of ELOVL3 is in agreement with the decreased concentration of very-long-acyl-chain ceramides and dihydroceramides in risperidone-treated cells. Regarding ELOV4, its activity catalyzes the first and rate-limiting reaction of the four reactions that constitute the ultra-long-chain fatty acid elongation cycle [24]. The increased expression of ELOVL4 could represent a compensatory response to the decreased synthesis of long-chain fatty acids. Weston-Green et al. reported that the SGA clozapine upregulated ELOVL1 protein and decreased ceramide and sphingomyelin levels in the liver of female rats [17]. Therefore, it may be suggested that during the treatment with antipsychotics, the expression of ELOVL isoforms is readapted as a consequence of the reduction of very-long-chain ceramides and dihydroceramides levels, which should be demonstrated by more direct studies.

Ceramide levels were lower in the plasma of older patients (mean age of 83 years) with vascular dementia than in healthy controls [33]. Lam et al. reported that the concentrations of sphingolipids, including very-long-chain ceramides, were decreased in postmortem brain tissues of subjects with vascular dementia [34]. Moreover, higher plasma ratios of very-long-chain to long-chain ceramides have been associated with a reduced risk of incident dementia and Alzheimer’s disease dementia in the Framingham offspring cohort, with a mean age of 70 years [35]. We cannot rule out that dementia increased the breakdown of sphingolipids in some tissues of risperidone-treated patients. Nevertheless, the decreased expression of ELOVL3 may contribute to the reduction in the levels of very-long-chain ceramides and dihydroceramides in these patients.

Antipsychotics are CADs, which are positively charged by virtue of an amine group that can be protonated, and have both hydrophilic and hydrophobic properties. CADs can cross biological membranes because of their amphiphilic character. As weak bases, CADs are protonated in the acidic environment of the lysosome and become trapped in this organelle [36]. One of the major concerns regarding the use of CADs is the induction of phospholipidosis, which is associated with the accumulation of the drug or its metabolites in multiple tissues [36]. Phospholipidosis is characterized by a decrease in lysophospholipid levels [37]. Saito et al. reported that the reduction in the lysophosphatidylcholine concentration was a potential blood biomarker for drug-induced hepatic phospholipidosis [38]. There were no detectable changes in total serum lysophosphatidylcholine between before and after 7 months of antipsychotic treatment of first-episode psychosis [19]. In turn, lysophosphatidylcholine levels were increased in the plasma of schizophrenia patients (average age of 30 years) after risperidone treatment for 2 to 3 weeks [15]. Our findings showed that risperidone treatment significantly decreased lysophosphatidylcholine concentrations in PBMCs and monocytes in vitro. Furthermore, we previously demonstrated that risperidone impaired intracellular lipid trafficking and induced lipid accumulation in endolysosomes [7]. Therefore, the decrease in lysophosphatidylcholines can be used as a marker of impaired intracellular lipid trafficking caused by risperidone.

In the present study, PBMCs were used as a peripheral model to explore what may be occurring in the brain. In this respect, it had been described by others that brain metabolism can be indirectly assessed by studying lipid composition in peripheral cells [39,40,41]. Therefore, changes in PBMCs can be considered as an approximation to what happens in the brain, although more direct studies are necessary.

In conclusion, we show that risperidone administration in older persons decreases the levels of dihydroceramides, very-long-chain ceramides, and lysophosphatidylcholines in circulating PBMCs. The levels of the latter lipid class could be used as a marker of intracellular lipid trafficking defects. Moreover, risperidone treatment disrupts lipid raft domains in the plasma membranes of PBMCs of older patients, potentially affecting multiple signaling pathways.

### Limitations

The sample size was small because of the difficulties in recruiting this kind of patient. Notwithstanding this, the findings were statistically significant and agreed with those obtained in a human monocyte cell line. In addition to the high age of our patients (mean age of 88.5 years), the similarities between the two groups of patients in terms of age and gender should be noted. It should also be mentioned that most of them were polymedicated due to the multiple pathologies they suffered because of their age. Although we did not find significant differences in co-medication between the groups, the effects of age or other drugs on treatment outcomes cannot be ruled out.

## 4. Materials and Methods

All chemicals, unless otherwise stated, were purchased from Sigma (Sigma-Aldrich Química, S.A., Tres Cantos, Madrid, Spain).

### 4.1. Participants

Two groups of patients older than 70 years of both sexes were recruited at the Geriatric Outpatient Clinic of the Hospital Universitario Ramón y Cajal, including patients not treated with antipsychotics or antidepressants for at least 3 months before the initiation of the study and without dementia or behavioral and psychological symptoms of dementia (*n* = 10) (control group), and patients with dementia or behavioral and psychological symptoms of dementia treated with risperidone at 0.5–3 mg per day for the 3 months before recruitment (*n* = 10) (treated group). The investigation conforms to the ethical guidelines of the Declaration of Helsinki. The study protocol was approved by the Research Ethics Committee of our hospital (Protocol No. 026/11). The exclusion criteria were treatment with statins or other lipid-lowering drugs, thiazides, valproate, estrogens, or anabolic drugs; renal insufficiency; liver disease with alanine transaminase (ALT), aspartate transaminase (AST), or total bilirubin more than twice the upper limit of normal serum concentrations; hypothyroidism or hyperthyroidism.

### 4.2. Biochemical Parameters

Serum samples were collected from fasting patients for hematological analysis. Biochemical parameters were analyzed using an ARCHITECT c16000 analyzer (Abbott, Madrid, Spain).

### 4.3. Isolation of PBMCs

Blood was drawn from fasting participants in test tubes containing lithium heparin, diluted 1:1 with PBS, and layered on Lymphoprep (Axis-Shield PoC AS, Oslo, Norway), as reported previously [42]. The samples were centrifuged at 800× *g* for 40 min and PBMCs were collected from the interphase and washed three times with PBS. The PBMC fraction was centrifuged, the supernatant was discarded, and the pellet was frozen at −80 °C until further analysis.

### 4.4. Cell Culture

Human THP-1 monocytes (ATCC TIB-202) were cultured in RPMI-1640 medium with L-glutamine (Fisher Scientific, S.L., Madrid, Spain), 0.05 mM 2-mercaptoethanol, 10% fetal bovine serum (FBS), and antibiotics at 37 °C in a humidified 5% CO_2_ atmosphere. Lipoprotein-deficient serum (LPDS) was prepared from FBS by ultracentrifugation at a density of 1.21 kg/L. The cells were cultured in medium with 10% LPDS in the presence or absence of 25 µM risperidone (Tocris, Bristol, UK) for 72 h as described before [7,11].

### 4.5. Lipidomic Analysis

Lipids were extracted from PBMCs (50–500 µg of protein) and THP-1 cells (400 µg of protein) according to Folch’s method [43]. The following internal standards were added for each lipid class before lipid extraction to obtain a relative molar quantification of lipid species: dihydroceramide (dhCer 35:0 (d18:0/17:0)) (Matreya LLC; State College, PA, USA), ceramide (Cer 37:1 (d18:1/19:0)), hexosylceramide (HexCer 33:1 (d18:1/15:0)), sphingomyelin (SM 30:1 (d18:1/12:0)), phosphatidylcholine (PC 28:2 (14:1:14:1)), phosphatidylethanolamine (PE 32:2 (16:1/16:1)), and lysophosphatidylcholine (LPC 17:1) (Avanti Polar Lipids; Alabaster, AL, USA). The lipid extracts were dried and suspended in 250 µL of acetonitrile/isopropanol (1:1). The lipid species were separated on a Kinetex C18 column (100 × 2.1 mm, 1.7 µm; Phenomenex, Macclesfield, UK) at 55 °C. Elution was carried out using a system consisting of solvent A (60% acetonitrile in water, 10 mM ammonium formate) and solvent B (90% isopropyl alcohol in acetonitrile, 10 mM ammonium formate), with a linear gradient from 60% A to 100% B in 12 min and 100% B to 60% A in 8 min at a flow rate of 0.4 mL/min. The lipid species were analyzed using mass spectrometry on a QTrap 4000 triple quadrupole (AB-Sciex LLP, Framingham, MA, USA) in positive ESI mode. Nitrogen was used as the drying gas at a temperature of 500 °C. The injection volume was 5 µL. Lipid species were identified by retention time and MS/MS fragmentation pattern, as previously described [44]. Briefly, ceramide and hexosylceramide species were detected by neutral loss scan (NLS) at *m*/*z* 264 and dihydroceramide was detected by NLS at *m*/*z* 266. Sphingomyelin, lysophosphatidylcholine, and phosphatidylcholine species were monitored by the loss of the phosphocholine head group at *m*/*z* 184 and phosphatidylethanolamine was detected by NLS at *m*/*z* 141. The LC-MS/MS peak chromatograms were processed using Skyline software version 4.1 [45], and molecular species were quantified by direct comparison of the area for each species with the area of the internal standard for their lipid class [46].

### 4.6. Analysis of Detergent-Resistant Membranes by Flow Cytometry

PBMCs (5 × 10^5^ cells) from the treated and control group were labeled with CD-13 FITC-conjugated antibody (EBioscience, Thermo Fisher Scientific, Waltham, MA, USA) for 20 min at room temperature. THP-1 cells were treated or not with 25 μM risperidone for 72 h and labeled as described above. The fluorescence intensity of CD13-FITC and FSC and SSC signals in labeled and control cells were measured using a flow cytometer before and 5 min after mixing the cells with 0.02% Triton-X100 on ice [22]. Ten thousand cells were acquired from each sample. The fluorescence signals were gated by marking a cell population on FSC-SSC dot plots and using the same marker for untreated and detergent-treated cells. Detergent resistance was estimated by the flow cytometric detergent resistance (FCDR) index according to the equation: FCDR = (FLT − AFUT)/(FLU − AFUU), where FLT and AFUT are the fluorescence of labeled detergent-treated cells and autofluorescence of unlabeled detergent-treated cells, respectively, and FLU and AFUU are the fluorescence of labeled untreated cells and autofluorescence of unlabeled untreated cells, respectively [22]. The FCDR value is low if a membrane protein is highly soluble after the addition of the detergent, while it is high (approaches 1) in the case of a typical detergent-resistant protein [22].

### 4.7. RNA Isolation and Quantitative RT-PCR (qRT-PCR)

Total RNA from THP-1 cells was extracted using TriReagent (MRC Molecular Research Center, Inc., Cincinnati, OH, USA) according to the manufacturer’s recommendations and reverse-transcribed with random hexamers using the PrimeScript RT reagent kit (Takara Bio Inc., Kusatsu, Shiga, Japan). The qRT-PCR amplification was performed on a LightCycler 480 using the SYBR Green I Master kit (Roche Applied Science, Penzberg, Germany). The amplification protocol consisted of an initial denaturation step at 95 °C for 5 min, followed by 45 cycles at 95 °C for 10 s, 60 °C for 10 s, and 72 °C for 10 s. Melting curves were analyzed and amplification products were separated on 2% agarose gels to confirm the presence of a single band. The efficiency of the reaction was evaluated by amplifying serial dilutions of cDNA (1:10, 1:100, 1:1000, and 1:10,000). We ensured that the relationship between the threshold cycle (Ct) and the log(RNA) was linear (−3.6 < slope < 3.2). All analyses were performed in triplicate and the expression of target genes was normalized to the housekeeping gene RPLP0 (encoding ribosomal protein large P0). The primers used in qRT-PCR are shown in Table S1.

### 4.8. Statistical Analysis

Data are shown as means ± SEMs. Differences in biochemical parameters, lipid profiles, and FCDR values between groups were analyzed using a *t*-test. Gene expression results were analyzed by one-way ANOVA analysis followed by Bonferroni post-hoc test. Means were considered significantly different when the *p*-value was less than 0.05.

## Figures and Tables

**Table 1:** Characteristics of study participants.

	Control (*n* = 10)	Risperidone (*n* = 10)
Age	88.5 ± 2.6	88.5 ± 2.3
Gender: male/female	5/5	5/5
Risperidone mean daily dose (mg)	—	1.05 ± 0.15
Self-caring (n)	6/10	1/10
Restricted diet (n)	0/10	0/10
Oral supplements (n)	0/10	1/10
Co-medication (n):		
Antidepressants	2/10	2/10
Anxiolytic, sedatives and hypnotics	1/10	3/10
Anti-dementia drugs	3/10	3/10
Anithypertensives	8/10	6/10
Anticoagulants	7/10	6/10
Diabetes drugs	2/10	2/10
Drugs used for respiratory diseases	5/10	3/10
Analgesics	7/10	8/10

**Table 2:** Biochemical parameters in serum of non-antipsychotic (control) and risperidone-treated (risperidone) older patients. Results are means ± SEMs, *n* = 10. Statistical comparisons shown are risperidone vs. control (* *p* < 0.05). ALT, alanine amino transferase; AST, aspartate amino transferase; γGT, γ glutamyl transpeptidase; TSH, thyroid-stimulating hormone.

	Control	Risperidone
Glucose (mg/dL)	100.3 ± 7.9	103.3 ± 14.7
Creatinine (mg/dL)	1.1 ± 0.2	0.8 ± 0.1
Uric acid (mg/dL)	5.6 ± 0.7	4.8 ± 0.8
Sodium (mM/L)	139.5 ± 1.4	138.7 ± 0.7
Potassium (mM/L)	4.2 ± 0.2	4.2 ± 0.2
Chlorine (mM/L)	104.2 ± 1.9	103.3 ± 1.1
Calcium (mg/dL)	8.8 ± 0.3	8.6 ± 0.2
Total protein (g/dL)	6.2 ± 0.3	5.6 ± 0.2
Total billirubin (mg/dL)	0.5 ± 0.1	0.5 ± 0.1
AST (U/L)	14.5 ± 1.6	21.7 ± 2.4 *
ALT (U/L)	11.5 ± 1.4	20.3 ± 3.8 *
γGT (U/L)	30.1 ± 6.1	23.4 ± 2.8
Lactate dehydrogenase (U/L)	163.2 ± 6.7	194.4 ± 28.6
Alkaline phosphatase (U/L)	79.9 ± 11.3	78.3 ± 5.9
C-reactive protein (mg/dL)	30.2 ± 13.4	85.7 ± 37.7
TSH (mU/L)	1.6 ± 0.2	2.5 ± 1.2
Total cholesterol (mg/dL)	159.5 ± 15.1	155.5 ± 20.5
Cholesterol-HDL (mg/dL)	33.9 ± 3.0	36.1 ± 6.9
Cholesterol-LDL (mg/dL)	99.6 ± 12.2	98.2 ± 14.0
Triglycerides (mg/dL)	128.4 ± 11.4	103.7 ± 16.7
Red blood cells (10^6^/µL)	3.8 ± 0.2	3.6 ± 0.3
Hemoglobin (g/dL)	11.5 ± 0.5	10.9 ± 0.9
Hematocrit (%)	33.7 ± 1.4	32.3 ± 2.6
Mean corpuscular volume (fl)	90.2 ± 2.0	90.6 ± 1.8
Platelets (10^3^/µL)	231.6 ± 35.8	249.1 ± 24.4
White blood cells (10^3^/µL)	8.1 ± 0.7	9.7 ± 1.3
Neutrophils (%)	68.7 ± 3.5	72.4 ± 5.2
Lymphocytes (%)	19.9 ± 3.0	19.2 ± 5.0
Monocytes (%)	8.3 ± 0.5	7.2 ± 1.0
Eosinophils (%)	2.8 ± 0.5	2.0 ± 0.3
Basophils (%)	0.4 ± 0.1	0.3 ± 0.1

## Data Availability

Data are contained within the article or Supplementary Material.

## Supplementary Materials

The following are available online at https://www.mdpi.com/article/10.3390/ijms22083919/s1.
